# Myricetin induces apoptosis and autophagy by inhibiting PI3K/Akt/mTOR signalling in human colon cancer cells

**DOI:** 10.1186/s12906-020-02965-w

**Published:** 2020-07-06

**Authors:** Ming-liang Zhu, Pei-min Zhang, Min Jiang, Shu-wen Yu, Lu Wang

**Affiliations:** 1 School of Pharmaceutical Sciences, Cheeloo College of Medicine, Shandong University, Jinan, 250012 China; 2 Department of Pharmacy, Jinan Central Hospital, Cheeloo College of Medicine, Shandong University, Jinan, 250013 China

**Keywords:** Myricetin, Autophagy, Apoptosis, Colon cancer, 3-MA

## Abstract

**Background:**

The compound 3,3′,4′,5,5′,7-hexahydroxyflavone (myricetin) is a natural flavonoid with antitumour activity. Most of the studies on myricetin have focused on the induction of tumour cell apoptosis, and little is known about the regulatory effects of myricetin on autophagy in colorectal cancer.

**Methods:**

Here, we studied the effects of myricetin on colon cancer cell proliferation, apoptosis and autophagy. We detected colon cancer cell apoptosis induced by myricetin via flow cytometry and Hoechst 33258 staining. Transmission electron microscopy was performed to observe the morphological changes associated with autophagy. The expression levels of apoptosis-, autophagy- and PI3K/Akt/mTOR signalling-related proteins were measured by Western blot analysis.

**Results:**

This study confirmed that myricetin inhibits the proliferation of 4 kinds of colon cancer cell lines. Myricetin induced cell apoptosis and autophagy by inhibiting PI3K/Akt/mTOR signalling pathway. In addition, the inhibition of autophagy with 3-methyladenine (3-MA) promoted the apoptosis of myricetin-treated colon cancer cells.

**Conclusions:**

Considering that myricetin induces apoptosis and autophagy in colon cancer cells, myricetin may become a viable candidate for chemotherapy; it could be used to exert tumour inhibitory effects alone or as adjuvant chemotherapy to inhibit autophagy. These studies may provide further evidence for the potential use of myricetin in the treatment of colon cancer.

## Background

Colorectal cancer is the third most common and second deadliest cancer worldwide, with an estimated 1.8 million new cases and 862,000 attributed deaths in 2018 [1]. Treatment of colorectal cancer is multifaceted and may include a combination of surgery, radiation therapy, chemotherapy, and targeted therapies such as checkpoint inhibitors and anti-angiogenesis therapies [2]. Despite improved treatment options, drug resistance exists for some patients receiving traditional chemotherapeutics [3]. For this reason, new chemotherapy drugs are needed in the clinic.

**Fig. 1:**
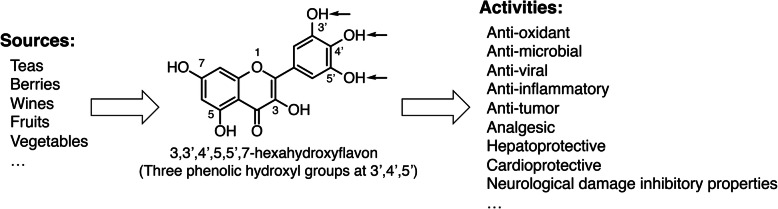
The source, chemical structure and activities of myricetin

Myricetin (3,3′,4′,5,5′,7-hexahydroxyflavone) is a natural flavonoid pigment commonly found in fruits, herbs, and nuts [4, 5]. Myricetin differs from other flavonols in the presence of the 3′,4′,5′-trihydroxy. Myricetin has been found to have anticancer properties in a variety of malignancies, including prostate [6], breast [7], gastric [8], and lung cancers [9] (Fig. 1). However, there are few studies on the role of myricetin in colorectal cancer. Kim et al. and Nirmala et al. showed that myricetin could inhibit the growth of colorectal cancer in vitro and in vivo [10, 11]. In 2018, Lee et al. suggested that myricetin selectively induces the apoptosis of HCT-15 colon cancer cells by increasing the expression of nucleoside diphosphate kinase and other caspase-regulated apoptosis proteins [12].

Apoptosis and autophagic cell death are two types of programmed cell death. An apparent conundrum is that autophagy acts in cytoprotection and in cell death. While these processes are different, there is a complex connection between apoptosis and autophagy [13]. Currently, there is no research on whether myricetin can induce autophagy in colorectal cancer cells. Therefore, we aimed to evaluate the role and mechanism of action of myricetin in the treatment of colorectal cancer. The findings demonstrated that myricetin could induce colorectal cancer cell apoptosis and autophagy and that the PI3K/AKT/mTOR signalling pathway might be involved in myricetin-induced cell proliferation, apoptosis and autophagy. In addition, inhibition of autophagy with 3-Methyladenine (3-MA) promoted myricetin-induced apoptosis of colorectal cancer cells.

## Methods

### Reagents

Myricetin, Hoechst Staining Kit (G3680) and resazurin were purchased from Solarbio Life Sciences (Beijing, China). 3-MA (S2767) was purchased from Selleck Chemicals (Houston, TX, USA). The primary antibodies included Bax (5023S), Bcl-2 (15071S), Microtubule-associated protein1 light chain 3 (LC-3) I/II (12741S), Beclin-1 (3495S), Phosphatidyl inositide 3-kinase (PI3K) (13666S), phospho-PI3K (4228S), phospho-Akt (4060S), Akt (2920S), Mammalian target of rapamycin (mTOR) (2983S), phospho-mTOR (5536S) were purchased from Cell Signaling (MA, USA). β-actin (60008–1) was purchased from ProteinTech Group (Wuhan, China). Horseradish peroxidase-conjugated secondary antibodies (ZB-2301, ZB-2305) were purchased from ZSGB-Bio (Beijing, China).

### Cell culture and treatment

Four human colorectal cancer cell lines, HT-29, HCT116, SW480 and SW620, were obtained from American Type Culture Collection (ATCC, Manassas, VA, USA). The cells were cultured in RPMI-1640 medium with 10% foetal bovine serum (Solarbio Science & Technology, Beijing, China) at 37 °C in a 5% CO_2_ atmosphere.

### Cell viability assay

Cell viability was measured with the resazurin cell viability assay. The cells were seeded at a density of 2 × 10^4^ cells/well in 96-well culture plates. After 4 h, different concentrations of myricetin (0, 12.5, 25, 50, 100, 200, and 400 μmol/L) were added and incubated for 24 h, 48 h, and 72 h. Cells incubated with culture medium with an equivalent amount of vehicle dimethyl sulfoxide (DMSO) served as controls. Next, 20 μL of deep blue-coloured resazurin solution (freshly prepared) was added to each group and incubated for 4 h. The absorbance was measured at excitation/emission wavelengths of 560/590 nm with a microplate reader to determine the cell survival rate [14].

### Hoechst 33258 staining

Colorectal cells were seeded at a density of 2 × 10^5^ cells/mL in 6-well plates. After fixation with 3.7% paraformaldehyde for 30 min at 37 °C, the cells were washed with phosphate-buffered saline (PBS) and treated with 10 mg/L Hoechst 33258 for 15 min. A fluorescence microscope was used to observe the colorectal cells. When cells are apoptotic, the chromatin will condense. Therefore, after Hoechst 33258 staining, under a fluorescence microscope, condensed apoptotic nuclei should have a strong fluorescence intensity increase and be smaller than normal nuclei. In addition, there were vesicles in the cell membrane and apoptotic bodies in apoptotic cells [15].

### Transmission electron microscopy

Autophagosome formation was detected using conventional electron microscopy using a previously established protocol [16]. First, the cells were fixed with 2.5% glutaraldehyde and 2.5% formaldehyde for 2 h before being incubated with 1% OsO_4_ for 1 h. Next, the cells were soaked in 2% uranyl acetate for 1 h and processed sequentially using acetone/Spur’s resin (1,1), ethanol, and 100% Spur’s resin. Transmission electron microscopy was used to visualize the ultrathin sections of cells.

### Western blot analysis

The colorectal cancer cells were inoculated into 6-well plates at a density of 4 × 10^5^ cells/mL and cultured at 37 °C overnight. Different concentrations of myricetin (25, 50, and 100 μmol/L) were added the next day, and a blank control group (0 μmol/L myricetin) was set up. The cells were collected after 48 h of incubation. Total cellular protein was extracted with radioimmunoprecipitation assay (RIPA) buffer. The proteins were separated on 10% sodium dodecyl sulfate (SDS)-polyacrylamide gels. After the bands were transferred to a polyvinylidene fluoride (PVDF) membrane, the membrane was blocked with 5% milk. Next, the membrane was incubated with the primary antibodies at 4 °C overnight. After washing three times with PBS for 10 min, the membrane was incubated with the secondary antibody for 45 min at room temperature [17].

### Flow cytometry assays

The cells were treated and collected as described above, and flow cytometry was performed using the Annexin V/Propidium iodide (PI) Apoptosis kit (Sigma, America). The cells were washed three times with PBS and resuspended in 1× binding buffer. Next, the cells were incubated in the dark with 5 μL of Annexin V-FITC and 10 μL of propidium iodide for 5 min each [18]. The samples were run on a FACSCalibur flow cytometer (BD Bioscience, San Jose, CA, USA), and the data were processed using FlowJo 10 (FlowJo, LLC, Ashland, OR, USA). Both early and late apoptotic cells were considered.

### Statistical analysis

Statistical analysis was performed by Student’s t-test for pair samples using SPSS/Win 13.0 software. The results are expressed as the mean ± SD, and significant outcomes are indicated as follows: *p < 0.05, **p < 0.01, and ***p < 0.001.

## Results

### Myricetin inhibited the viability of human colorectal cancer cells

**Fig. 2:**
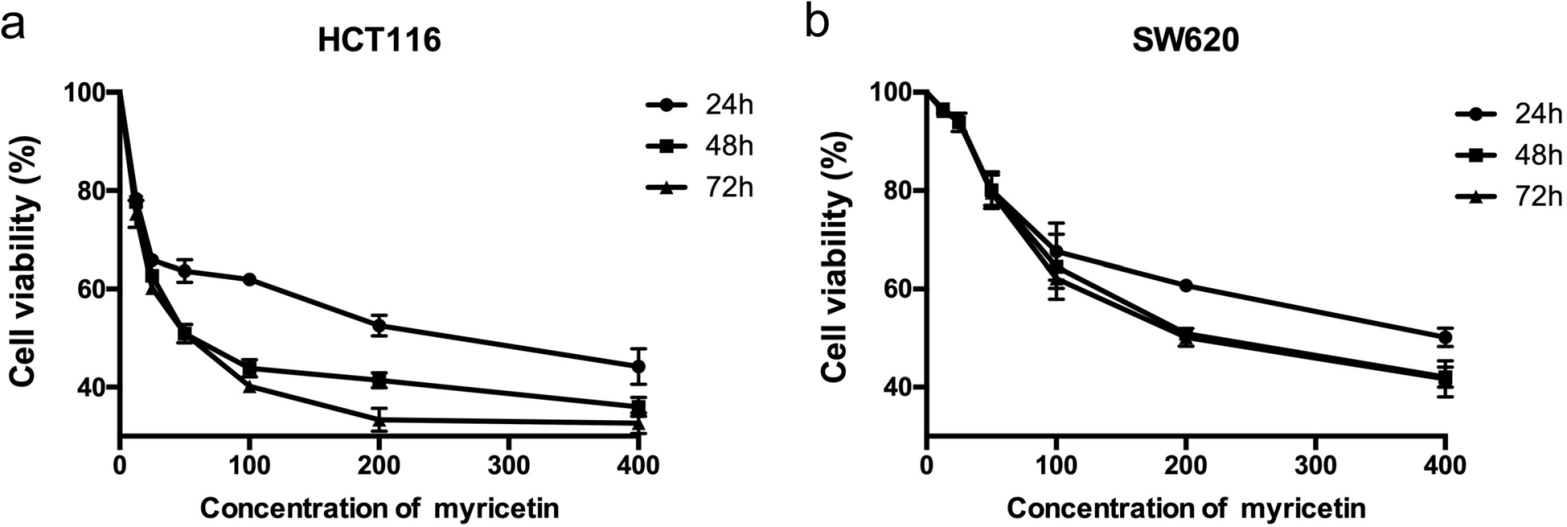
Myricetin inhibited the viability of human colorectal cancer cells. HCT116 and SW620 cells were treated with 0–400 μmol/L myricetin for 24 h, 48 h, and 72 h. Cell viability was analysed by means of a resazurin cell viability assay. Three replicate wells were set up in 96-well plates for each experimental group, and the experiment was repeated three times

We investigated the effects of myricetin on four different human colorectal cancer cell lines (HT-29, HCT116, SW480, SW620) by means of a resazurin cell viability assay. Colorectal cells were treated with 6 concentrations of myricetin (12.5, 25, 50, 100, 200 and 400 μmol/L) for 48 h. The results showed that HCT116 and SW620 cells were more sensitive to myricetin than the other two cell lines, and their IC_50_ values were 83.45 and 233.4 μmol/L, respectively (Table 1). Therefore, these two cell lines were selected for subsequent experiments. Myricetin inhibited the proliferation of HCT116 and SW620 cells in a dose-dependent manner. Since there was no significant difference in cell survival between the 48 h and 72 h treatments with myricetin, 48 h was selected for the subsequent experiments (Fig. 2).

**Table 1:** The growth inhibitory effect of myricetin against different human colon cancer cell lines expressed as a percentage

Conc.(μmol/L)	HCT116	HT-29	SW480	SW620
	Inhibition(% ± SE)			
12.5	22.310 ± 0.633	2.057 ± 0.532	3.507 ± 0.289	3.440 ± 0.662
25	37.323 ± 0.752	3.443 ± 0.749	6.160 ± 0.346	6.103 ± 1.075
50	49.086 ± 1.093	19.733 ± 0.687	19.223 ± 0.901	19.933 ± 2.149
100	56.146 ± 1.002	25.710 ± 2.785	26.957 ± 3.165	35.507 ± 3.813
200	58.570 ± 0.900	37.253 ± 2.428	43.710 ± 2.197	49.070 ± 0.563
400	64.030 ± 1.109	52.070 ± 3.371	55.197 ± 3.599	57.947 ± 1.169
Regression equation	y = 11.52ln(x)-1.134	y = 14.74ln(x)-39.37	y = 14.28ln(x)-36.20	y = 17.19ln(x)-44.53
R^2^	0.9215	0.9677	0.9762	0.9772
IC50(μmol/L)	83.45	350.4	291.3	233.4

### Myricetin induced apoptosis of HCT116 and SW620 cells

**Fig. 3:**
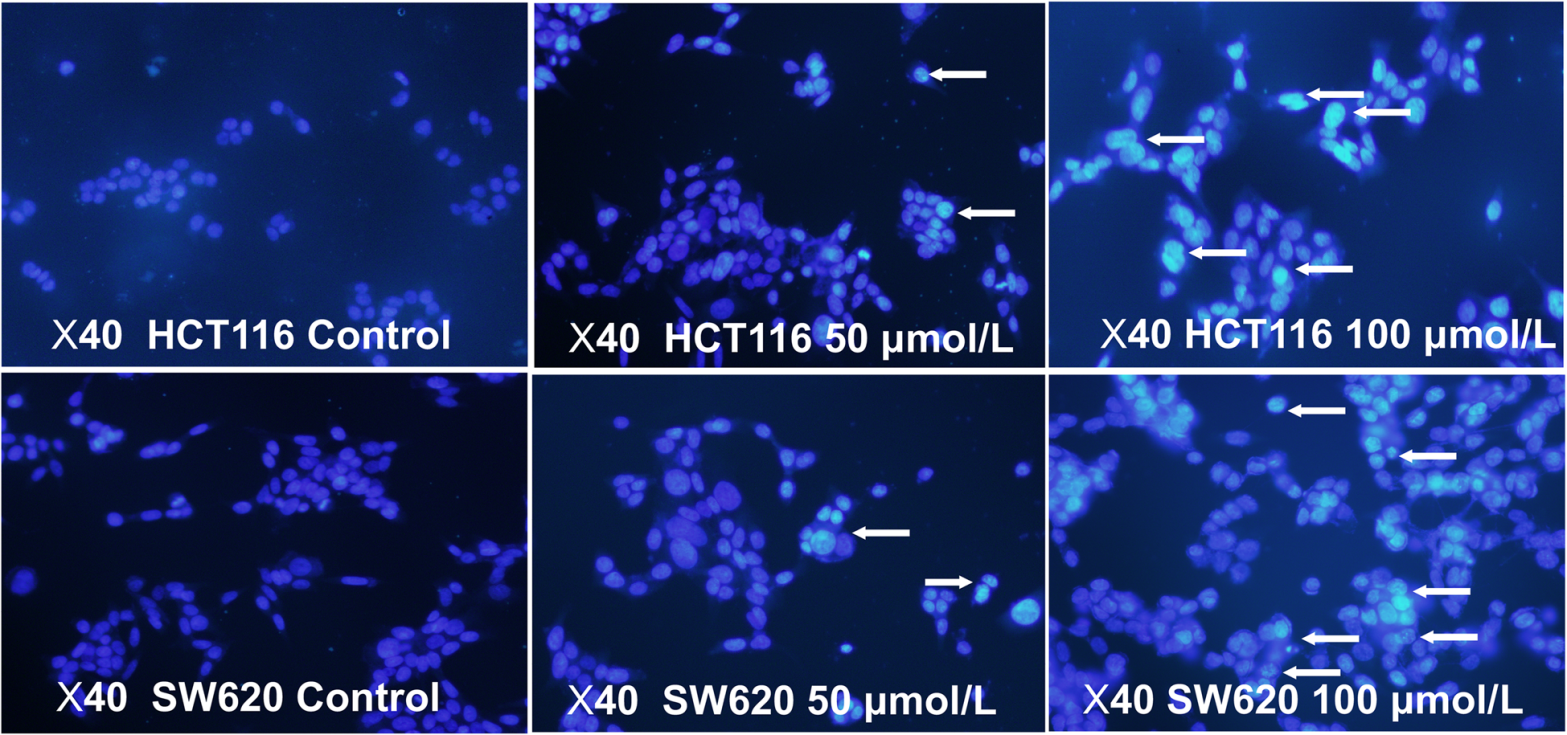
Myricetin induced the apoptosis of HCT116 and SW620 cells. Hoechst 33258 staining was used to detect apoptosis, indicated by arrows (× 200 magnification). Cells were treated with 50 or 100 μmol/L myricetin for 48 h

**Fig. 4:**
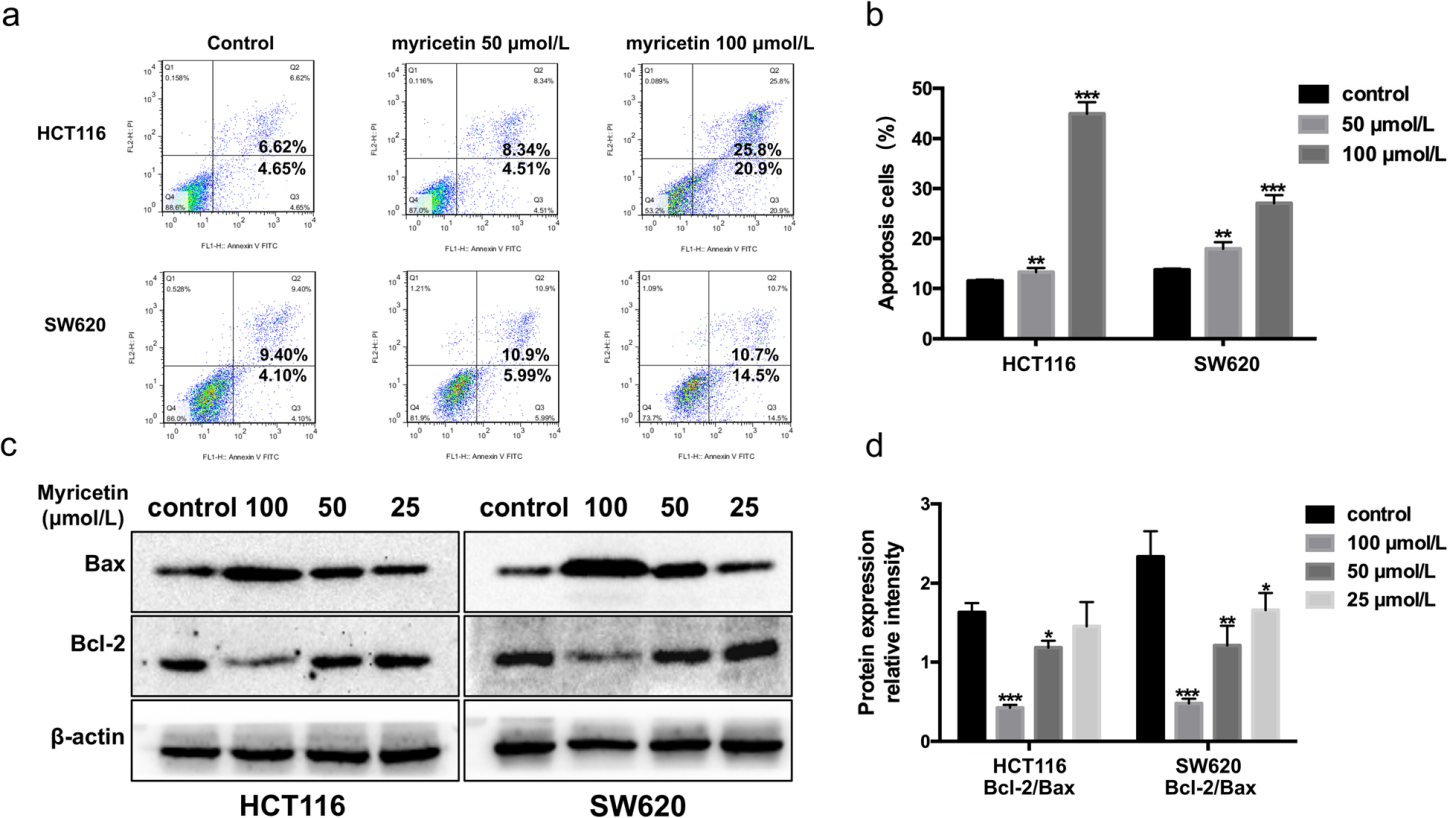
Myricetin induced the apoptosis of HCT116 and SW620 cells. **a** Apoptosis was determined by flow cytometry, and **b** the percentage of cell viability was quantified with a histogram (the early and late apoptotic cells are also considered). **c** Bax and Bcl-2 were detected by Western blotting with β-actin as the loading control. **d** Quantification of the Western blotting results as a histogram. The experiment was repeated three times (*p < 0.05, **p < 0.01, and ***p < 0.001 vs. control)

Here, we verified the effects of myricetin on the apoptosis of colon cancer cells via Hoechst 33258 staining. Hoechst 33258 staining is a commonly used method for visualizing apoptosis by observing chromatin condensation under a fluorescence microscope. Apoptotic cells were positively stained with fluorochrome Hoechst 33258 (Fig. 3). In colorectal cancer cells treated with 50 and 100 μmol/L myricetin for 48 h, light microscopy showed that the apoptotic cells became rounder and smaller. In addition, there were vesicles in the cell membrane and apoptotic bodies in the cell. The percentage of apoptotic cells was 28.5 and 67.4% in the 50 and 100 μmol/L myricetin-treated groups, respectively. The number of apoptotic cells was positively correlated with the concentration of myricetin. Annexin V-FITC/PI double stain flow cytometry assay was used to determine the rate of cell apoptosis [19]. Flow cytometry revealed similar findings, with the apoptotic rate being positively correlated with the concentration of myricetin (Fig. 4a-b). The results from the Western blotting assay further validated that myricetin reduced the ratio of Bcl-2/Bax and induced apoptosis in a dose-dependent manner. As the concentration of myricetin increased, the Bax content in the cells increased, and the Bcl-2 content decreased (Fig. 4c-d). All of the above experiments showed that myricetin could induce the apoptosis of colon cancer cells.

### Myricetin induced autophagy by inhibiting PI3K/Akt/mTOR signalling in HCT116 and SW620 cells

**Fig. 5:**
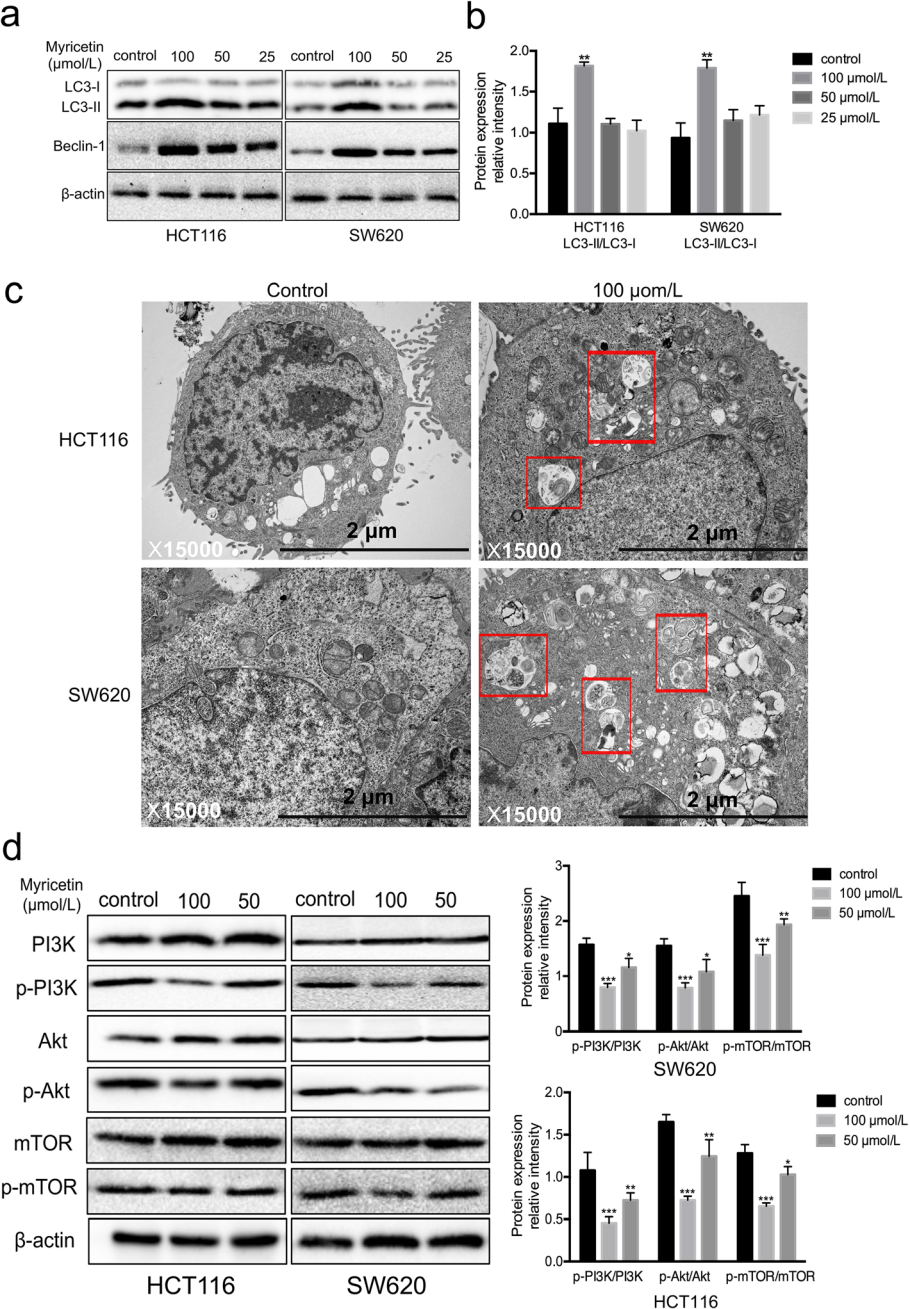
Myricetin induced autophagy by inhibiting the activation of the PI3K/Akt/mTOR signalling pathway in HCT116 and SW620 cells. **a** LC3-I/II and Beclin-1 were detected by Western blotting with β-actin as the loading control. **b** Quantification of the Western blotting results with a histogram. **c** Transmission electron microscopy showed the number of autophagosomes (rectangle) in the cells treated with myricetin. **d** Protein levels were detected by Western blotting with β-actin as the loading control. Cells were treated with 50 or 100 μmol/L myricetin for 48 h. The Western blots were analysed by densitometry in ImageJ, and the myricetin treatment group was compared with the control group. The experiment was repeated three times (*p < 0.05, **p < 0.01, and ***p < 0.001 vs. control)

Previous studies have shown that myricetin can induce apoptosis of HCT-15 and HT-29 human colon cancer cells [11, 12, 20, 21], and Cao J et al. in 2018 reported that myricetin can induce autophagy in HepG2 liver cancer cells [22]. However, the effect of myricetin on autophagy in colon cancer cells is still unknown. During the formation of autophagosomes, LC3-I transforms into LC3-II, resulting in a gradual increase in LC3-II expression [23]. Simultaneously, the expression of Beclin-1, which acts as a key induction gene in the autophagy process, also increases [24]. In the current study, Western blot analysis showed that the ratios of LC3-II/β-actin and Beclin-1/β-actin expression were increased in HCT116 and SW620 cells in a dose-dependent manner after myricetin treatment (Fig. 5a-b). In addition, electron microscopy revealed an increase in the number of autophagosomes in the cells treated with myricetin compared to those that were not treated (Fig. 5c). The percentage of autophagic cells was 73.1% in the myricetin-treated group. Together, these findings verify that myricetin induces autophagy in human colon cancer cells.

Western blot analysis was used to detect the protein expression levels of phospho-PI3K, PI3K, phospho-Akt, Akt, phospho-mTOR, and mTOR in the HCT116 and SW620 cell lines. While phospho-PI3K, phospho-Akt, and phospho-mTOR were decreased in a dose-dependent manner, there was no significant difference in the total PI3K, Akt, or mTOR. The ratios of p-Akt/Akt and p-mTOR/mTOR were significantly decreased following myricetin treatment (Fig. 5d). These data suggest that myricetin induces cell apoptosis and autophagy partly through inhibition of the PI3K/Akt/mTOR signalling pathway.

### Inhibition of autophagy enhanced myricetin-induced apoptosis in human colon cancer cells

**Fig. 6:**
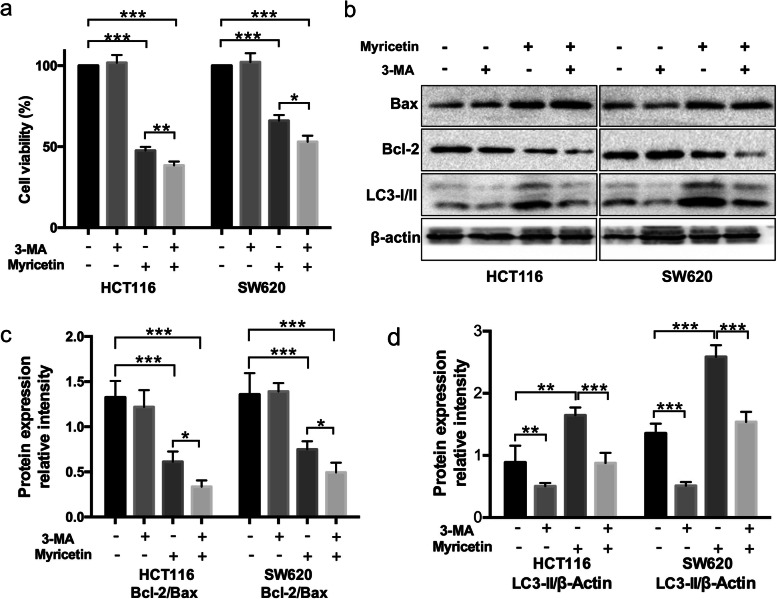
Inhibition of autophagy enhanced apoptosis induced by myricetin in HCT116 and SW620 cells. **a** Cells, with or without pre-treatment with 3-MA for 1 h, were treated with 100 μmol/L myricetin for 48 h. Cell viability was investigated with a resazurin cell viability assay. **b**-**d** The Western blots show densitometry by ImageJ, and the myricetin treatment group was compared with the control group. Bax, Bcl-2, and LC3-I/II were measured in cells following treatment with 100 μmol/L myricetin for 48 h, with or without pre-treatment with 3-MA for 1 h. The experiment was repeated three times (*p < 0.05, **p < 0.01, and ***p < 0.001)

HCT116 and SW620 cells, with and without pre-treatment with the autophagy inhibitor 3-MA for 1 h, were treated with 100 μmol/L myricetin for 48 h. 3-MA was dissolved in RPMI-1640 medium (the final concentration was 5 mM). Cell viability was assessed via a resazurin cell viability assay (Fig. 6a), and the protein content was detected by Western blot analysis (Fig. 6b). Western blot analysis showed that the LC3-II/β-actin ratio decreased in the cells that were pre-treated with 3-MA compared to those that were not pre-treated, suggesting that autophagy was effectively inhibited by 3-MA treatment. In addition, cell viability was obviously reduced in HCT116 and SW620 cells that were pre-treated with 3-MA for 1 h when compared with cells that were not pre-treated with 3-MA. Western blot analysis also showed increased Bax content and decreased Bcl-2 content in the 3-MA pre-treated HCT116 and SW620 cells, suggesting that autophagic inhibition by 3-MA treatment enhanced myricetin-induced apoptosis in colorectal cancer cells (Fig. 6c-d).

## Discussion

Chinese herbal medicine has been shown to improve immune function, promote phagocytosis of the reticuloendothelial system, protect the haematopoietic function of bone marrow and inhibit decreases in white blood cells and platelets [25, 26]. In recent years, an increasing number of traditional Chinese herbal medicines have been used in the adjuvant treatment of cancer. Myricetin, a natural flavonoid, is widely distributed in many natural plants, such as the bark of *Myrica nagi* Thunb and the leaves of *Ampelopsis sinica* (Miq.) W.T. Wang and *Camellia sinensis* (L.) Kuntze [27, 28]. Most studies on the antitumour mechanism of myricetin have focused on its roles in inducing apoptosis and inhibiting inflammation, revealing its potential role in the progression of cell growth. In the present study, the effects of myricetin on the induction of colon cancer cell apoptosis and autophagy were investigated. Myricetin exhibited anti-colon tumour activities that were mediated by apoptotic and autophagic pathways.

The PI3K/AKT/mTOR pathway is an intracellular signalling pathway that is important in regulating the cell cycle. Therefore, it is directly related to cellular quiescence, proliferation, cancer, and longevity [29, 30]. Phillips PA reported that myricetin induced pancreatic cancer cell death via the induction of apoptosis and inhibition of the PI3K signalling pathway [31]. The PI3K/AKT/mTOR signalling pathway might be involved in the regulation of autophagy and might affect cell proliferation [32–34]. In our study, Western blotting was used to detect the different signalling pathways in HCT116 and SW620 cells treated with myricetin, and the results demonstrated that myricetin-induced cell apoptosis and autophagy were mediated by the PI3K/AKT/mTOR signalling pathway.

Apoptosis and autophagy, two distinct modes of cell death, are both involved in the inhibitory effects of myricetin in colon cancer. In past reports, both autophagy and apoptosis have been found to occur in the same cells [35, 36]. Autophagy and apoptosis are reported to intersect with each other via mTOR and AMPK signalling [37, 38]. The AMPK and Bax signalling pathways have been demonstrated to activate autophagy [39], and numerous studies have revealed autophagy-induced apoptosis [40, 41]. Nevertheless, in recent years, some reports have shown that the inhibition of autophagy can increase the sensitivity of cancer cells to chemotherapeutic drugs by reducing drug resistance [42, 43]. It is generally believed that autophagy plays a dual role in the development of cancer. In the early stages of cancer, autophagy inhibits the growth of cancer cells. However, autophagy can also promote the development of tumours through a protective mechanism during later stages of cancer [44]. In our study, by inhibiting autophagy with 3-MA, we found that myricetin increased the apoptosis of human colorectal cancer cells. Hence, inhibition of autophagy promoted myricetin-induced apoptosis of colorectal cancer cells. The protective effect of autophagy is also an important mechanism by which colon cancer cells develop resistance to myricetin. Considering that myricetin itself inhibits colon cancer cell proliferation and induces apoptosis, the combination of myricetin and autophagy inhibitors may be a viable option for chemotherapy or adjuvant chemotherapy. In-depth studies of the mechanism of myricetin are still needed in the future.

## Conclusions

Myricetin has been found to have anticancer properties in a variety of malignancies. However, the exact mechanisms of these effects are not fully known. There are few studies on the efficacy of myricetin as an anticancer agent in colon cancer, and little is known about the regulatory effects of myricetin on autophagy. The findings from the present study suggest that myricetin induces colon cancer cell autophagy and apoptosis by inhibiting PI3K/Akt/mTOR signalling. In addition, the inhibition of autophagy increases the apoptosis of myricetin-treated colon cancer cells. Therefore, myricetin may become a viable candidate for chemotherapy; it could be used alone to exert tumour inhibitory effects or as adjuvant chemotherapy to inhibit autophagy. These studies may provide further evidence for the potential use of myricetin in the treatment of colon cancer. The findings lay the basis for developing more efficient anti-colon cancer drugs derived from natural products. Future research is needed to develop highly effective antitumour drugs that target cancer cells.

## Data Availability

The datasets used and/or analysed during the current study are available from the corresponding author on reasonable request.

## Abbreviations

MA: 3-methyladenine
LC3: Microtubule-associated protein 1 light chain 3
PI3K: Phosphatidyl inositide 3-kinase
mTOR: Mammalian target of rapamycin
ATCC: American Type Culture Collection
DMSO: Dimethyl sulfoxide
PBS: Phosphate-buffered saline
RIPA: Radio immunoprecipitation assay
SDS: Sodium dodecyl sulfate
PVDF: Polyvinylidene fluoride
FITC: Fluorescein isothiocyanate
PI: Propidium iodide
